# Assessment the Trend of Inequality in the Distribution of Intensive Care Beds in Iran: Using GINI Index

**DOI:** 10.5539/gjhs.v6n6p28

**Published:** 2014-06-24

**Authors:** Mohammad Meskarpour-Amiri, Parisa Mehdizadeh, Mohsen Barouni, Nooredin Dopeykar, Maryam Ramezanian

**Affiliations:** 1 Health Economics Department, Health Management Research Center, Baqiyatallah University of Medical Sciences, Tehran, Iran; 2 Health Economics Department, Faculty of Management and Economics, Tarbiat Modares University, Tehran, Iran; 3 Master of Sciences in Health Economics, Hospital Management Research Center, Iran University of Medical Sciences, Tehran, Iran; 4 Assistance Professor, Research Center for Health Services Management, Institute for Futures Studies in Health, Kerman University of Medical Sciences, Kerman, Iran; 5 Research Center for Modeling in Health, Institute for Futures Studies in Health, Kerman University of Medical Sciences, Kerman, Iran; 6 Research Center for Health Services Management, Institute for Futures Studies in Health, Kerman University of Medical Sciences, Kerman, Iran

**Keywords:** inequality, Intensive Care Beds, GINI Index, ICU, CCU, NICU

## Abstract

**Background and Aim:**

While most of the published researches have reported the amount of inequity in geographical distribution of important health resources, only a small number of studies have focused on the trend of inequality in the distribution of these resources.

The purpose of this study was to determine the trend of inequality in the distribution of intensive care beds in Iran during 2010 to 2012 by using the Gini coefficient.

**Methods:**

This is a cross-sectional research conducted in 2013. The changes over three years (2010 to 2012) were calculated by Gini coefficient to investigate the trend of inequality in geographical distribution of intensive care beds (CCU, ICU and NICU).

**Results:**

The Gini coefficient for CCU beds was calculated as 0.02, 0.04 and 0.06 in 2010, 2011 and 2012, respectively. The Gini coefficient for ICU beds was calculated as 0.03, 0.05 and 0.05 in 2010, 2011 and 2012, respectively. Also, the Gini coefficient for NICU bed was calculated as 0.02, 0.03 and 0.04 in 2010, 2011 and 2012, respectively.

**Conclusion:**

Regarding to Gini coefficient, the trend of inequality was increased in the distribution of intensive care beds in Iran. Particularly, the inequalities in distribution of CCU beds were significantly increased during past years. In fact, if this trend of inequality continues, the distribution of intensive care beds will be extremely unequal in the next five years in Iran.

## 1. Background

In general, one of the most important goals of a health system is to provide public accessibility and equality in receiving health and treatment services (Kiadaliri, Najafi, & Haghparast-Bidgoli, 2011; Balarajan, Selvaraj, & Subramanian, 2011). Since accessibility to health is one of the basic rights of individuals in the society; nowadays accessibility of individuals in a society to health services counts as one measure of development (Kottke & Isham, 2010; Mackenbach et al., 2008). Furthermore, limited accessibility to health services and the inequality in availability of the services furnished by health and treatment systems deprives these people of acceptable and effective treatment (Kottke & Isham, 2010; Reddy, 2004; Tofighi, Meskarpour, & Ameryoun, 2011).

Inequality in health services in different countries has taken the form of a global challenge (Mackenbach et al., 2008; Tang et al., 2008) affected by various factors, including individual, social and geographical variables (Mackenbach et al., 2003; Marmot, 2001). These factors are stronger in developing countries (Balarajan et al., 2011) so much so that the geographical distribution of health in developing countries has turned into a fundamental issue. Therefore, the measurement of fair distribution of health services on top of previous measures has been emphasized by World Health Organization (World Health Organization, 2000).

Identifying the number and types of intensive care beds and their distribution may prove as an indirect measurement of the accessibility to inpatient intensive care services (Horev, Pesis-Katz, & Mukamel, 2004; Pedersen & Lilleeng, 2000). Despite the fact that in many cases the proportion of beds to population may be used as a measure of the distribution of health services, a high proportion does not necessarily imply an equal accessibility of the population to such facilities and services. Therefore, examining the distribution of a service in a given geographical location could be a supplementary criterion to the existing measures of central tendencies (Horev et al., 2004).

Iran, like other developing countries, is faced with the lack of intensive care resources (Ameryoun, Meskarpour-Amiri, Lorgard Dezfuli-Nejad, Khoddami-Vishteh, & Tofighi, 2011; Tofighi, Meskarpour Amiri, Ameriuon, & Naseri, 2011). Although the number of intensive care beds has grown substantially in recent years, it does not still seem enough. Previous studies (Ameryoun et al., 2011) showed that the number of per population intensive care beds in Iran is lower than those in many developing countries. Shortage of per population intensive care beds in Iran causes problems such as patient transference between provinces (Ameryoun et al., 2011), long waiting line of receiving intensive care unit services (Abedi, Seiyamiyan, & Rostami, 2012) increase mortality and cost of treatment (Ameryoun et al., 2011; Tofighi et al., 2011), reducing the patient satisfaction (Abdi, Delgoshaei, Ravaghi, Abbasi, & Heyrani, 2013) and quality of care (Falahinia, Zareian, Oshvandi, Farhanchi, & Moghimbigi, 2013) in intensive care units. One solution to these problems is to increase the number of intensive care beds in short term, but according to the limited resources this doesn’t seem rational and feasible. Another solution is to distribute the available intensive care beds more efficiently and equitably. In terms of intensive care beds shortage, the uneven distribution of the bed can greatly threaten the access to care. In other words, inequality in the distribution of intensive care beds could increase the problems caused by the shortage of this beds. Thus, the distribution of intensive care beds in developing countries like Iran seems to be more important.

In Iran, establishment of public hospitals by government and especially set up of intensive care units in public hospitals are greatly influenced by two main factors, the number of population and the bargaining power of local politicians. Therefore, the needs of population and regional epidemiology of diseases have received lower attention in health resource allocation. Additionally, the activities of the private sector in health care were always based on the principle of profit; however the government`s incentives were not useful to encourage private sector to develop in less developed areas (Ameryoun et al., 2011; Tofighi et al., 2011).

While many studies have focused on measuring the amount of inequality in the distribution of health resources in both developed and developing countries, there still is a question here: whether the distribution of health care resources goes toward equality or inequality? In fact, most of the published researches have emphasized on the inequality in geographical distribution of important medical resource such as hospital beds (Ameryoun et al., 2011; Mackenbach et al., 2008; Nishiura et al., 2004), Medical staffs (Fülöp, Kopetsch, Hofstätter, & Schöpe, 2008; Kreng & Yang, 2011; Theodorakis & Mantzavinis, 2005) and medical equipment (Chun-xia, Lang, Yu-ming, & Zhao-hui, 2007; He Yu, & Chen, 2013), however only a small number of studies have focused on the trend of inequality in the distribution of these resources.

Inequality in receiving health services is measured by different scales (Williams & Doessel, 2006), one of which is the Gini coefficient which is based on the Lorenz curve. This coefficient compares the cumulative frequency of the distribution of a given variable with the normal distribution of that variable (which shows equality) (Berndt, Fisher, & Rajendrababu, 2003; Williams & Doessel, 2006).

Over the past few years, dramatic growth has been occurred in the number of intensive care beds in Iran. Only from 2010 to 2011, the number of intensive care beds has increased by 12.6%. Due to the increase in intensive care beds in Iran, the attention of policy makers and researchers have been on this question that whether the trend in distribution of intensive care beds is towards equality or inequality? The purpose of this study was to determine the trend of inequality in the distribution of intensive care beds in Iran during 2010 to 2012, using the Gini coefficient.

## 2. Materials and Methods

This study was a cross-sectional research that performed in 2013. Changes in the Gini coefficient over three years (2010 to 2012) were used to study the trend of inequality in geographical distribution of intensive care beds (CCU, ICU and NICU). The population data of Iranian provinces from 2010 to 2012 were obtained from the Statistics Center of Iran. The number of intensive care beds (ICU, Post ICU and NICU beds) in each province from 2010 to 2012 was based on the latest reported information of the Ministry of Health and Medical Education of Iran. According to the prior studies (Ameryoun et al., 2011; He et al., 2013), the Gini coefficient is calculated by the following formula:


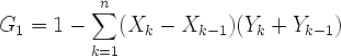


Where X was the cumulative percentage of the population and Y was the cumulative percentage of each type of intensive care beds. The Gini coefficient ranges between 0 and 1, where theoretically, zero corresponds to a perfect equality and 1 corresponds to a perfect inequality. Based on the previous studies (Ameryoun et al., 2011; He et al., 2013), Gini coefficient which is smaller than 0.2 is considered as low inequality level; between 0.2 and 0.3 is considered as moderate inequality; between 0.3 and 0.4 is considered as high inequality; higher than 0.4 is considered as extreme inequality.

The demographic data as well as the number of CCU, ICU and NICU beds were entered into MS Excel spreadsheet. The number of each type of CCU and Post CCU bed per 100.000 people, and the cumulative percentages of each type of bed were calculated. Finally, the Gini coefficients for each type of bed were calculated by using the above formula.

## 3. Results

In 2010, 2011 and 2012, Iran had a total population of 73.650.566, 74.733.231 and 75.149.669 with total numbers of 3160, 3430 and 3665 CCU beds, the total number of 3384, 3576 and 3698 ICU beds and the total number of 1189, 1295 and 1349 NICU beds, respectively. Tehran, the capital of Iran, had the largest amount of CCU, ICU and NICU beds and population during 2010-2012. The total numbers of CCU, ICU and NICU beds from 2010 to 2012 are shown in Table 1.

**Table 1:** Total number of CCU, ICU and NICU beds from 2010 to 2012 by province

Province	2010				2011				2012			
		
	Population	CCU	ICU	NICU	Population	CCU	ICU	NICU	Population	CCU	ICU	NICU
East Azerbaijan	3667968	125	157	83	3691270	143	162	86	3724620	159	176	88
West Azerbaijan	2979604	105	146	52	3016301	110	147	56	3080576	110	147	57
Ardebil	1238778	40	79	18	1242956	42	81	18	1248488	44	83	18
Isfahan	4741615	224	222	75	4804458	235	232	76	4879312	238	232	76
Ilam	561001	16	18	8	566332	17	19	9	557599	18	19	10
Bushehr	928930	45	35	17	943535	46	37	17	1032949	46	41	17
Tehran	14448184	717	790	247	12505705	763	780	256	12183391	871	801	273
Karaj	NE	NE	NE	NE	2289412	66	45	10	2412513	66	48	10
Chaharmahal&Bakhtyari	883856	30	34	20	892909	32	34	20	895263	34	34	21
South Khorasan	666493	26	23	14	676794	27	26	17	662534	27	38	19
RazaviKhorasan	5852010	149	208	119	5940766	153	231	129	5994402	155	237	131
North Khorasan	831684	25	25	20	838781	25	25	20	867727	25	25	20
Khuzestan	4420874	154	164	29	4471488	165	168	30	4531720	179	179	38
Zanjan	978310	25	52	19	983369	29	67	20	1015734	34	67	22
Semnan	615601	37	53	14	624482	37	53	14	631218	37	53	14
Sistan&Baluchestan	2650767	103	111	57	2733205	119	123	67	2534327	119	123	67
Fars	4479087	280	241	55	4528514	284	248	64	4596658	287	252	66
Qazvin	1194771	23	29	10	1212464	26	32	10	1201565	28	32	10
Qom	1107145	40	74	20	1127713	40	74	24	1151672	40	74	24
Kordestan	1460180	62	34	14	1467585	70	47	19	1493645	70	47	19
Kerman	2872902	122	87	52	2947346	126	108	57	2938988	138	117	61
Kermanshah	1898464	70	116	45	1905793	73	119	45	1945227	70	119	45
Kohkiluyeh & Buyer Ahmad	660216	15	16	6	669140	15	16	15	658629	15	16	15
Golestan	1669019	78	65	17	1687086	92	65	20	1777014	92	69	24
Gilan	2440405	82	61	13	2453469	95	70	13	2480874	109	74	13
Lorestan	1747159	93	82	29	1758226	113	84	40	1754243	117	84	43
Mazandaran	3007570	166	221	69	3037336	178	230	69	3073943	222	234	69
Markazi	1381645	66	50	26	1392435	66	53	26	1413959	66	53	26
Hormozgan	1519700	66	34	8	1558878	67	37	11	1578183	68	40	14
Hamedan	1699815	49	82	17	1699588	49	85	21	1758268	54	99	23
Yazd	1046816	127	75	16	1065893	127	78	16	1074428	127	85	16
Total	73650566	3160	3384	1189	73733231	3430	3576	1295	75149669	3665	3698	1349

NE: Not exist (after the Parliamentary approval, Karaj was introduced as 31st province of Iran from 2011).

There were 4.29, 4.59 and 4.87 CCU beds for each 100.000 people across the nation in 2010, 2011 and 2012 respectively. There were 4.59, 478 and 4.92ICU beds for each 100.000 people across the nation in 2010, 2011 and 2012 respectively. Also, there were 1.61, 1.73 and 1.79 NICU beds for each 100.000 people in 2010, 2011 and 2012 respectively. Table 2 shows the number of CCU, ICU and NICU beds per 100.000 populations from 2010 to 2012.

**Table 2:** Number of CCU, ICU and NICU beds per 100.000 population from 2010 to 2012

Province	2010			2011			2012		
		
	CCU	ICU	NICU	CCU	ICU	NICU	CCU	ICU	NICU
East Azerbaijan	3.408	4.28	2.263	3.874	4.389	2.33	4.269	4.725	2.363
West Azerbaijan	3.524	4.9	1.745	3.647	4.874	1.857	3.571	4.772	1.85
Ardebil	3.229	6.377	1.453	3.379	6.517	1.448	3.524	6.648	1.442
Isfahan	4.724	4.682	1.582	4.891	4.829	1.582	4.878	4.755	1.558
Ilam	2.852	3.209	1.426	3.002	3.335	1.589	3.228	3.407	1.793
Bushehr	4.844	3.768	1.83	4.875	3.921	1.802	4.453	3.969	1.646
Tehran	4.963	5.468	1.71	6.101	6.237	2.047	7.149	6.575	2.226
Karaj	NE	NE	NE	2.883	1.966	0.437	2.736	1.99	0.415
Chaharmahal&Bakhtyari	3.394	3.847	2.263	3.584	3.808	2.24	3.798	3.798	2.346
South Khorasan	3.901	3.451	2.101	3.989	3.842	2.512	4.075	5.736	2.868
RazaviKhorasan	2.546	3.554	2.033	2.575	3.888	2.171	2.586	3.954	2.185
North Khorasan	3.006	3.006	2.405	2.981	2.981	2.384	2.881	2.881	2.305
Khuzestan	3.483	3.71	0.656	3.69	3.757	0.671	3.95	3.95	0.839
Zanjan	2.555	5.315	1.942	2.949	6.813	2.043	3.347	6.596	2.166
Semnan	6.01	8.609	2.274	5.925	8.487	2.242	5.862	8.396	2.218
Sistan&Baluchestan	3.886	4.187	2.15	4.354	4.5	2.451	4.696	4.853	2.644
Fars	6.251	5.381	1.228	6.271	5.476	1.413	6.244	5.482	1.436
Qazvin	1.925	2.427	0.837	2.144	2.639	0.825	2.33	2.663	0.832
Qom	3.613	6.684	1.806	3.547	6.562	2.128	3.473	6.425	2.084
Kordestan	4.246	2.328	0.959	4.77	3.203	1.295	4.687	3.147	1.272
Kerman	4.247	3.028	1.81	4.275	3.664	1.934	4.695	3.981	2.076
Kermanshah	3.687	6.11	2.37	3.83	6.244	2.361	3.599	6.118	2.313
Kohkiluyeh& Buyer Ahmad	2.272	2.423	0.909	2.242	2.391	2.242	2.277	2.429	2.277
Golestan	4.673	3.895	1.019	5.453	3.853	1.185	5.177	3.883	1.351
Gilan	3.36	2.5	0.533	3.872	2.853	0.53	4.394	2.983	0.524
Lorestan	5.323	4.693	1.66	6.427	4.778	2.275	6.67	4.788	2.451
Mazandaran	5.519	7.348	2.294	5.86	7.572	2.272	7.222	7.612	2.245
Markazi	4.777	3.619	1.882	4.74	3.806	1.867	4.668	3.748	1.839
Hormozgan	4.343	2.237	0.526	4.298	2.374	0.706	4.309	2.535	0.887
Hamedan	2.883	4.824	1	2.883	5.001	1.236	3.071	5.631	1.308
Yazd	12.13	7.165	1.528	11.91	7.318	1.501	11.82	7.911	1.489
Total	4.291	4.595	1.614	4.59	4.785	1.733	4.877	4.921	1.795

NE: Not exist (after the Parliamentary approval, Karaj was introduced as 31st province of Iran from 2011).

**Figure 1:**
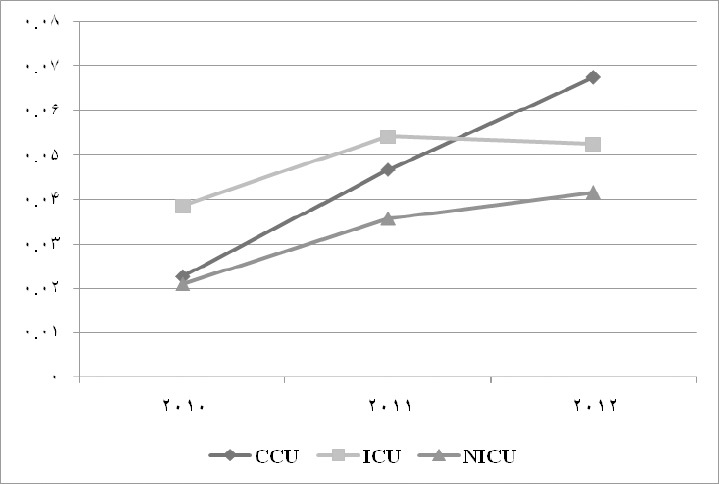
Trend of Gini coefficient of CCU, ICU and NICU during 2010-2012

The Gini coefficient for CCU bed was calculated as 0.02, 0.04 and 0.06 in 2010, 2011 and 2012 respectively. The Gini coefficient for ICU bed was calculated as 0.03, 0.05 and 0.05 in 2010, 2011 and 2012 respectively. Also, the Gini coefficient for NICU bed was calculated as 0.02, 0.03 and 0.04 in 2010, 2011 and 2012 respectively. Table 3 shows the Gini coefficient for CCU, ICU and NICU beds from 2010 to 2012. Also, Figure 1 illustrates the trend of inequality in geographical distribution of each intensive bed during 2010-2012.

**Table 3:** Gini coefficient for CCU, ICU and NICU from 2010 to 2012

Gini coefficient	2010	2011	2012
CCU	0.0227	0.0468	0.0676
ICU	0.0386	0.0542	0.0526
NICU	0.0210	0.0358	0.0416

## 4. Discussion

Generally speaking, it should be noted that the cost-effectiveness of the establishment of intensive care units is much less than the cost-effectiveness of preventive measures such as immunization and primary health care. Therefore, based on the principle of prioritizing resource allocation, especially in the developing countries like Iran, it is necessary to consider the cost-opportunity of investment in the development of intensive care units. The shortage of intensive care beds in Iran has been cited in numerous studies (Abedi et al., 2012; Ameryoun et al., 2011; Tofighi et al., 2011). However, this study was intended to examine the issue of inequality trend in the distribution of existing intensive care beds in Iran regardless of whether or not a shortage exists in number of such beds. As a matter of fact, the unequal distribution of intensive care beds often leads to unequal distribution of valuable health resources such as specialist doctors, medical equipment and trained nurses.

The findings show that the mean number of CCU beds per 100.000 population in 2010, 2011 and 2012 are 4.29, 4.59 and 4.87, respectively. In each of the three years, Yazd province with 12.13, 11.91 and 11.82 beds per 100.000 population had the highest CCU beds than any other provinces (two times more than that of other provinces). Also, Qazvin province in 2010 and 2011 with 1.92 and 2.14 respectively and Kohkiluyeh Province in 2012 with 2.27 had the lowest CCU beds per 100.000 population. The obtained Gini coefficient for the distribution of CCU beds in 2010, 2011 and 2012 was 0.022, 0.046 and 0.067 respectively which statically proves the equality in geographical distribution of CCU beds across Iran. But the trend of Gini coefficient during the three years was toward inequality, So that the numerical value of the Gini coefficient was nearly tripled from 2010 to 2012.

In a study, Kiadaliri et al. (Kiadaliri, Safari, & Hosseinpour, 2010) showed that Yazd province enjoys the most equal distribution of CCU beds and Ilam province suffers from the least equal distribution of CCU beds. A study on the distribution of active hospital beds in Iran, the Gini coefficient for active beds was reported 0.08 and active beds per 10.000 population in 2006 was reported 9.2, while Yazd province had maximum and Lorestan province had the minimum beds per population (Tofighi, Maleki, Shahabi, Delpasand, & Nafisi, 2010). In another study conducted in 2002 based on Morris Imbalance Coefficient, Yazd province stands at the highest rank in terms of the number of hospital beds and Semnan province ranks the highest in terms of the number of health centers (Eskandari, 2010). A study conducted on the distribution of CCU beds in 24 university hospitals in the Netherlands from 2004 to 2006, Gini coefficients were reported 0.638, 0.569 and 0.569, which reflect the unequal distribution (De Bruin, Bekker, & Van Zanten, 2010).

The mean number of ICU beds per 100.000 population in 2010, 2011 and 2012 are 4.59, 4.78 and 4.92, respectively. In each of the three years, Semnan province with 8.60, 8.48 and 8.39 beds per 100.000 population had the highest ICU beds than any other provinces. Also, Hormozgan province in 2010 with 2.23 and Alborz province (Karaj) in 2011 and 2012 with 1.96 and 1.99 respectively had the lowest ICU beds per population. The obtained Gini coefficient for the distribution of ICU beds in 2010, 2011 and 2012 was 0.038, 0.054 and 0.052 respectively; as a result it proves the equality in geographical distribution of CCU beds across Iran. However, the general trend of Gini coefficient during the three years was toward inequality, so that the numerical value of the Gini coefficient has generally been rising from 2010 to 2012.

In the same study conducted by Ameryoun et al. (2011) in 2011, the number of ICU beds per 100.000 population in Iran in 2006 was reported 5.3 and the Gini coefficient was reported 0.17. Also, Semnan with 8.6 ICU beds per 100.000 population considered as the second province with the maximum of ICU beds. In comparison with the results of our study, the mean number of ICU beds is much more than the obtained Gini coefficient, since according to Ameryoun et al. (2011) the reported number of ICU beds was belonged to both public and private sectors. In a study on the distribution of ICU beds in Western Europe countries, Wunsch et al. (Wunsch et al., 2008) showed that the proportion of ICU beds per 100.000 people in 2005 was 9.3 in France, 8.4 in the Netherlands, 8.2 in Spain and 5.3 in England. In South Africa, there were 4, 168 ICU beds in 2005, from which 86% were installed in three provinces. The proportion of beds varied greatly in different provinces of Iran, from 1:20.000 to 1:80.000 (Bhagwanjee & Scribante, 2007). A study conducted by Horev et al in the U.S. in 1998 on the distribution of hospital beds, the coefficients in different states of America were 0.0571-0.4303 (Horev et al., 2004). The 1970-1997 trends indicated the progressive equality in the distribution of hospital beds. It has been reported that the northern states enjoy an equal distribution of hospital beds (Horev et al., 2004).

Also, the mean number of NICU beds per 100.000 population in 2010, 2011 and 2012 are 1.61, 1.73 and 1.79, respectively. In each of the three years, Northern Khorasan province with 2.40, 2.38 and 2.30 beds per 100.000 population had the highest NICU beds than any other provinces (two times more than that of other provinces). In addition, Hormozgan province in 2010 with 0.52 and Alborz provience (Karaj) in 2011 and 2012 with 0.43 and 0.41 respectively had the lowest per population NICU beds. The obtained Gini coefficient for distribution of NICU beds in 2010, 2011 and 2012 was 0.021, 0.035 and 0.041, respectively which statically proves the equality in geographical distribution of CCU beds across Iran. However, the general trend of Gini coefficient during the three years was toward inequality, so that the numerical value of the Gini coefficient was about double from 2010 to 2012. In the same study which was conducted in Iran, the number of NICU beds per 100,000 population and Gini coefficient were reported 1.6 and 0.23, respectively (Ameryoun et al., 2011).

The high prevalence and morbidity associated with cardiovascular diseases is one of the most pressing health problems in Iran. The findings of the studies showed that the prevalence of cardiovascular diseases in Iran is higher than the prevalence of cardiovascular diseases in Western countries and some Middle East countries (Ebrahimi, Kazemi-Bajestani, Ghayour-Mobarhan, & Ferns, 2011; Talaei et al., 2013). According to this dilemma, therefore, it is necessary to pay more attention on appropriate distribution of intensive care beds in Iran.

However there are some limitations in this study including that in this study we examined the distribution of intensive care beds in the public sector only, so with considering the private sector the inequality may be more because the private sector is often developed by the demand and ability to pay not population needs. Also recommended that in future studies the equity in distribution of intensive care beds examined based on the epidemiology of diseases and specific needs of each province.

## 5. Conclusion

According to our study, although in all years (2010-2012) the numerical value of Gini coefficients proves equality in distribution of Intensive Care Beds across Iran but the trend of Gini coefficients was toward inequality. In other words, increasing rate of inequality observed in Gini coefficient trend for Intensive care beds in Iran. Particularly the inequalities in distribution of CCU beds are significantly increased during past years. If this trend of inequality did persist, in the next five years the distribution of intensive care beds in Iran can be extremely unequal.
